# Supplementary material for: Estimating diabetes mellitus incidence using health insurance claims data: A database-driven cohort study
Source: PLoS One. 2024 Oct 2;19(10):e0311517. doi: 10.1371/journal.pone.0311517 (PMC11446451; doi:10.1371/journal.pone.0311517)
Supplement: Attachment — Submitted filename: Response_to_Reviewers20240807.docx [file pone.0311517.s002.docx]

Response to Reviewers

Dear Hamid Reza Baradaran and Reviewers,

Thank you for your suggestions on improving our manuscript.

As to restrictions on data sharing, the submission form includes the full information required as the following.

*a) A description of the data set and the third-party source*

*b) If applicable, verification of permission to use the data set*

*c) Confirmation of whether the authors received any special privileges in accessing the data that other researchers would not have*

*d) All necessary contact information others would need to apply to gain access to the data*

“The data generated and analyzed during this study cannot be shared publicly, due to the Ethical Guidelines for Medical and Biological Research Involving Human Subjects established jointly by Japanese ministries. Contracts were signed with the municipalities from which the data was provided, including restrictions on data users. However, other researchers may send data access requests to Office of Research Promotion, General Affairs and Planning Division, Kyoto University (E-mail: [060kensui@mail2.adm.kyoto-u.ac.jp)](mailto:060kensui@mail2.adm.kyoto-u.ac.jp)).”

Reviewer #1

1-1. *If the authors can provide some additional discussion about representativeness of the data of the city to support understandings of readers to know about the meaning of the data under comparison with some other references such as NDB open Data.*

Making comparative references between this study and the NDB Open Data, which are currently provided as relatively simple aggregate data, was difficult. Instead, we have added a reference for the differences between the handling of cross-sectional data, such as NDB Open Data, and the longitudinal data.

“While cross-sectional analyses are often challenging, longitudinal analyses present even greater difficulties.”

1-2. *Additional information about legal limitation of data sharing about NDB data should be denoted into the Acknowledgement part.*

The NDB, which we have cited in the document, has strict legal restrictions on using and sharing data, but this study did not use NDB. Therefore, we did not add any further references beyond the current text.

Reviewer #2

2-1. *Please include the approval number/ethics code indicating approval of this research in the ethics statement.*

We added the approval number.

2-2-1. *Line 46, page 3: The reference (2) cites the 2nd term of Health Japan 21, while in the text authors mentioned the 3rd term. Also, the URL link provided in the reference list directs to a page with a “404 Not Found” error. Please consider correcting the citation.*

This was the authors’ English translation mistake. “The third term”’ is correct.

The URL is correctly listed in our manuscript, but the PDF generator of the submission system caused an error.

*2-2-2. Throughout the manuscript the phrase “prevalence rate” is used, which is incorrect. Prevalence is a proportion, not a rate (because rates have a specified period of time in their denominator which prevalence lacks) and unfortunately, it is a common mistake the authors make in the scientific literature. So please correct all of the “prevalence rate” phrases to simply “prevalence” in the abstract, main body, tables, and figures. Also, this applies to diabetes medication usage and insulin usage rates.*

Thank you for your guidance. We changed “prevalence rate” to “prevalence” throughout the manuscript.

*2-2-3. Line 48, page 3: It is a fact that cross-sectional studies report prevalence and it is not necessary to cite an article for this regard. Also, the cited reference is not supporting the statement. Please consider removing the citation or adding more related citations.*

We removed this citation.

*2-2-4. Line 52, page 3: The cited reference reported the incidence of type II diabetes in Japan as 8.8 (95% CI: 7.4–10.4) per 1000 person-years. Please check the reference and correct it accordingly.*

Based on the cited literature, in addition to the CIs, we showed that their estimates are heterogeneous. We revised the manuscript to clarify this issue.

“approximately 8.8 (95% CI: 7.4–10.4) per 1000 person-years in Japan, although large variations in the original data were observed (2.3–52.6).”

*2-2-5. Line 53, page 3: Please update the URL link provided in reference (5) as it redirects to a page with the error “The page you're looking for was not found”.*

We updated this URL.

*2-2-6. Line 58, page 3: Please spell out the full term at its first mention, indicate its abbreviation in parenthesis, and use the abbreviation from then on.*

We made this revision.

“the NDB database (National Database of Health Insurance Claims and Specific Health Checkups of Japan),”

*Methods:*

*2-2-7. Please clearly mention the study design in the Methods section.*

We made this revision.

“This is a data-driven retrospective cohort study utilizing health insurance claims data from the National Health Insurance and medical care system of Saga City.”

*2-2-8. The words “ledger”, “reimbursement”, and “claim” can have various meanings in different countries based on their legal, fiscal, and medical systems. It would be great if you provide the exact meaning of these words according to the Japanese systems and laws in the Methods section.*

To clarify the notations concerning claims, we unified the terms to “claim,” replacing “reimbursement” with “claim,” and added a description of “claim” and “ledger.”

“A health insurance claim in Japan is an invoice used by healthcare institutions to bill insurers and other responsible parties for the portion of medical costs not covered by the patient. A breakdown of medical or prescription fees being claimed is included in the invoice.”

“The ledger data records the insured persons held by the insurer and also includes insured persons who do not use healthcare.”

*2-2-9. In the Methods section, Please clearly explain how you calculated the population at risk for each fiscal year.*

We made this revision.

*2-2-10. Please indicate which medications you considered as diabetes medications.*

We made this revision.

“medication (drugs with the first three digits of the Japanese drug price reference code 396, listed in Supporting Information 1)”

*2-2-11. Results:Please report the annual incidence data without grouping for age and sex (i.e. in Total for all ages and all sexes). Also, please consider statistically comparing the age groups and sexes with each other for significant differences.*

The annual incidence for all of the categories was added. Although differences between categories could be tested, this would require multiple tests, so we decided not to report the differences between categories. However, an additional statistical analysis was performed and reported to account for these groups, as suggested by the editor and other reviewers.

“The overall annual incidence, without categorization by sex or age, was estimated at 3.03%. The model, accounting for age and sex, also estimated the rate at 3.03% (95% CI: 2.21%–3.85%).”

*2-2-12. In the Results section please mention the exact numbers/percentages if you did not mention them in full in the tables or figures.*

The exact numbers are marked on the table.

*2-2-13. Please mention the number of the included and excluded individuals in the study. You can consider drawing a flowchart.*

In this study, simple inclusion was made from the database, as indicated in the Methods, and no exclusions were made.

*2-2-14. Line 105, page 6: In addition to fiscal year and age group, data are also grouped by sex, please add it to title of the Table 1. Line 112, page 7: Like Table 1, please correct Table 2’s title and add age group and sex.*

We made this revision.

*2-2-15. Line 115, page 7: You mentioned PR as the “change” in prevalence. Considering the fiscal year (FY) 2015 as the reference year, then there should not be a column dedicated to FY 2015. I highly recommend replacing this table with a similar table that shows the annual incidence rate by age group, FY, and sex.*

This was a mistake. “Prevalence by FY from FY2015” is correct and the present table is appropriate.

*2-2-16. Line 117, page 8: If Table 3 shows the annual incidence rate, then it should present the data for each year, if it shows the mean annual rate, then you should mention this both in the title and text. Also, Table 3’s lines are faded and not visible.*

As shown in the Methods, this is the calculation of slopes of the increased prevalence. We added an explanation to the Table 3.

“Annual incidence is calculated as the slopes of the increases in prevalence”

*2-2-17. According to the figure titles*

We made this revision.

*Discussion:*

*2-2-18. In the first paragraph of the Discussion section, please only summarize the main findings.*

We made this revision.

*2-2-19. I do not think it is a good idea to compare the findings of a city in Japan to the national incidence rate of South Korea considering significant differences between them. It will be more suitable if you compare your findings to the articles reporting rates for other municipalities and prefectures in Japan, or even at the national level so you can assess the comparability of your findings with territories sharing more similar context.*

I agree. We retained the quotation but rewrote the text to clarify that the comparison of figures was not meaningful.

“Although these rates were higher than the previously reported rates (i.e., 9.5–9.8/1000 person-years) in South Korea [5], direct comparisons between these two regions may not be meaningful due to significant differences in their underlying environments.”

*2-2-20. The second paragraph of the Discussion section (lines 139-145) is very unclear and hard to understand. Please consider rewriting it.*

We revised the text.

Thank you very much for your detailed feedback that allowed us to improve our manuscript.

Reviewer #3:

3-1.*The abstract lacks sufficient detail about the methods.*

A model analysis was added and the methods were revised.

*3-2.In the main text, there is inadequate information on data validity, the primary data source, and the data collection process.*

We revised the methods explaining the data source.

“This is a data-driven retrospective cohort study utilizing health insurance claims data from the National Health Insurance and medical care system of Saga City. The dataset is formally created and operated. A copy of this dataset was provided for research use under an agreement dated April 1, 2020.”

*3-3.While they included data from a proportion of participants under 75 years, the selection process and potential selection bias were not addressed.*

The data did not represent a partial selection of people aged 74 years and under but represented the limited number of citizens who have this insurance. We believe that this did not affect the results, but we have added the description of this point as a limitation.

“One limitation of this study is that the data are not all-inclusive for the entire population. A proportion of residents under 75 years of age are included. The main reason for the loss of follow-up during the study period was changes in insurance, including moving. Of note, our analysis included cases of mortality occurring during the study period, suggesting that the actual prevalence and incidence rates may be higher. While this study primarily focused on relatively straightforward analyses, more complex approaches, such as survival analysis, may also be considered, depending on the study’s context and objectives. Although we believe that the results will not be significantly distorted by these limitations, we cannot ensure that the figures are definitive based on this study alone. However, the methodology is useful.”

*3-3. My primary concern lies in the analysis: why was a Poisson regression model not utilized to assess the effects of age group, sex, and year? They report incidence and prevalence by these variables without indicating any interactions among them. I strongly recommend modeling the data.*

We acknowledge the suggestion to use a Poisson regression model. However, after careful consideration, we decided to employ a generalized linear model as it better suits the nature of our study. We have added this analysis:

“Finally, a generalized linear model weighted by the denominator was constructed to analyze the annual increase in prevalence, considering age, sex, and their interaction (R 4.4.1).”

Reviewer #4:

4-1. *Abstract: I think it is necessary to show the confidence intervals of the estimated measures as a proxy for precision of the estimates.*

A model analysis and confidence intervals have been added to the abstract.

“The overall annual incidence was estimated at 3.03% (95% CI: 2.21%–3.85%)”

4-2. *Keywords: OK; may be “big data” is not appropriate keyword for this study*.

We removed “big data” from the Keywords.

4-3. *Introduction: Please show what NDB stands for. It is also necessary to express the novelty or applicability of the study in this section explicitly*.

We added an explanation of the abbreviation “NDB,” and the text was revised to clarify that this study is challenging because tracking the data at the individual level was difficult in contrast to national NDB.

“the NDB database (National Database of Health Insurance Claims and Specific Health Checkups of Japan)”

4-4. *Material and method: Please notice to the following issues in this section:
- Please define the nominator and denominator used for estimating the proposed measures in detail in this section.*

We revised the explanation by mentioning the denominator and numerator.

“The denominator for each year was calculated using the constant number of cases in the population in 2015 for both years, whereas the numerator for each year was the number of cases for each year.”

*4-5. It is necessary to address loss to follow up for estimating the incidence rate.*

We added this point as a limitation to the study.

*4-6. The method of data analysis has been missed.*

We added a model analysis and revised the methods.

*4-7. Another issue to be noticed is the effect of new people entering to the proposed age groups and the mortality rate.*

This study covers individuals who can be tracked for 5 years. There were no transfers from the middle of the study period. The text was revised to make this explicit. Conversely, deaths during the period were included in the population analysis, which could introduce bias. This was added as a limitation of the study.

“Prevalence, utilization of diabetes medication (drugs with the first three digits of the Japanese drug price reference code 396, listed in Supporting Information 1), and insulin utilization were calculated by 5-year age groups per FY based on their age in FY2015. Subsequent analyses included insured individuals who could be tracked for 5 years from FY2015 to FY2019. Individuals who died within this period were also included. The continuity of individual persons was determined using claims data and ledger data for the insured persons. The ledger data records the insured persons held by the insurer and also includes insured persons who do not use healthcare.”

“The main reason for the loss of follow-up during the study period was changes in insurance, including moving. Of note, our analysis included cases of mortality occurring during the study period, suggesting that the actual prevalence and incidence rates may be higher. While this study primarily focused on relatively straightforward analyses, more complex approaches, such as survival analysis, may also be considered, depending on the study’s context and objectives.”

*4-8. As the last point, I think the validity of the method shall be addressed in this section as well.*

The main considerations were included in the discussion, but we have also mentioned this point in the methods section.

“This straightforward method corresponds to the slope of the graph for prevalence up to each FY”
