# Supplementary material for: Estimating diabetes mellitus incidence using health insurance claims data: A database-driven cohort study
Source: PLoS One. 2024 Oct 2;19(10):e0311517. doi: 10.1371/journal.pone.0311517 (PMC11446451; doi:10.1371/journal.pone.0311517)
Supplement: Attachment — Submitted filename: Response_to_Reviewers20240909.docx [file pone.0311517.s003.docx]

Response to Reviewers

Dear Hamid Reza Baradaran and Babak Eshrat,

Thank you for your suggestions on improving our manuscript.

*please define the method of denominator estimation in more detail.*

*As one the reviewers has mentioned please define the method of denominator estimation in more detail.*

*Reviewer #4: I think it is necessary to define the method of denominator estimation in more detail. This is especially true considering emigration or death which ordinarily happens in populations.*

Thank you for your comment. In the methods section, we originally stated that the relevant analysis was based solely on individuals who could be tracked for 5 years, which is a simple and straightforward approach without additional conditions. In the discussion, we noted that individuals who left the insurance system, such as those who moved, were excluded by this method. However, as per your suggestion, we have now added a mention of those exclusions in the methods section for clarity.

“Subsequent analyses included insured individuals who could be continuously tracked for 5 years from FY2015 to FY2019, excluding those who left the insurance system for reasons such as moving or had gaps in their tracking data, while still including those who died during this period.”

*Reviewer #4: Please define the method of confidence interval estimation.*

We used R and used a standard generalized linear model with weights and computed confidence intervals based on the standard errors of the coefficients. We made this revision.

“Finally, a generalized linear model weighted by the denominator was constructed to analyze the annual increase in prevalence, considering age, sex, and their interaction (R 4.4.1). Confidence intervals for the coefficient was estimated using standard errors derived from the weighted model.”

There was one instance in the previous revision where a terminology update was missed, which may have caused some confusion. We have now ensured consistency in the terms related to the confidence interval.

“The model, accounting for age and sex, also estimated the annual incidence at 3.03% (95% CI: 2.21%–3.85%).”
